# Tumour-stroma ratio and prognosis in gastric adenocarcinoma

**DOI:** 10.1038/s41416-018-0202-y

**Published:** 2018-07-30

**Authors:** Niko Kemi, Maarit Eskuri, Anni Herva, Joni Leppänen, Heikki Huhta, Olli Helminen, Juha Saarnio, Tuomo J. Karttunen, Joonas H. Kauppila

**Affiliations:** 1 Cancer and Translational Medicine Research Unit, Medical Research Center, University of Oulu and Oulu University Hospital, Oulu, Finland; 2 Upper Gastrointestinal Surgery, Department of Molecular Medicine and Surgery, Karolinska Institutet, Karolinska University Hospital, Stockholm, Sweden

**Keywords:** Cancer microenvironment, Gastric cancer

## Abstract

**Background:**

Tumour microenvironment, including tumour–stroma ratio (TSR), might help identifying high-risk cancer patients. However, the significance of TSR in gastric cancer is unclear, especially in the intestinal and diffuse subtypes. The aim of this study was to investigate the tumour–stroma ratio in gastric adenocarcinoma, and its intestinal and diffuse histological subtypes, in relation to prognosis.

**Methods:**

Five hundred and eighty-three gastric adenocarcinoma patients who underwent surgery in Oulu University hospital during years 1983–2016 were included in this retrospective cohort study. TSR was analysed from the slides that were originally used for diagnostic purposes. Patients were divided into stroma-poor (≤50% stroma) and stroma-rich (>50% stroma) groups and TSR was analysed in relation to 5-year mortality and overall mortality.

**Results:**

Patients with stroma-rich tumours had worse 5-year prognosis (HR 1.80, 95% CI 1.41–2.28) compared to stroma-poor tumours. Stratified analysis showed that stroma-rich tumours had worse 5-year prognosis in both intestinal (HR 1.68, 95% CI 1.24–2.27) and diffuse histological types (HR 2.09, 95% CI 1.35–3.23) compared to stroma-poor tumours, respectively.

**Conclusions:**

High proportion of stroma is an independent prognostic factor in both intestinal and diffuse histological subtypes of gastric adenocarcinoma.

## Introduction

Gastric cancer is the fifth most common malignancy and the third most common cause of cancer-related death worldwide^1^. Prognosis remains poor despite the development in treatments during the last decades, with 5-year mortality of 29%^2^. Gastric adenocarcinomas are classified with TNM classification based on depth of infiltration and number of local lymph node- and distal metastases. However, some patients with early T-stage without lymph node- or distant metastases still have poor outcomes. Therefore, it would be important to find new factors to recognise such high-risk patients that would benefit the most from aggressive adjuvant therapies after surgery.

Stromal component of tumour has been proven to greatly impact the development of the tumour^3^. Cancer-associated fibroblasts (CAFs) that produce the components of desmoplastic stroma seem to be very important in this regard^4^. The amount of stroma on haematoxylin–eosin (HE)-stained sections of a tumour can be easily assessed without additional stainings, and its assessment is potentially clinically applicable. Tumour–stroma ratio (TSR) is defined as the proportion of tumour cells relative to surrounding stroma, and it has been recognised as a potential prognostic factor for various solid tumours^5^. Few previous studies with limited sample size have suggested that gastric cancers with high proportion of stroma (low TSR) have poorer prognosis compared to gastric cancers with low proportion of stroma (high TSR)^6–9^. However, the relevance of TSR in the different histological types of gastric adenocarcinoma is poorly known, especially in the Western populations.

The aim of this study was to evaluate the reproducibility of the assessment of TSR and to elucidate the association between TSR and prognosis in gastric adenocarcinoma and its histological subtypes in a Western cohort.

## Materials and methods

### Study design

This study was a retrospective cohort study in a single institution in a tertiary care hospital in Northern Finland. There were 601 patients who underwent gastrectomy for gastric cancer in Oulu University Hospital between years 1983 and 2016. Five hundred and eighty-three of those were gastric adenocarcinoma patients whose diagnostic tissue sections were available for analysis and were included in the study.

### Data collection

The patients were identified from the archives of the Department of Pathology at the Oulu University Hospital, Finland. Clinical data for each patient were obtained from patient records, including operation charts and pathology reports. For each patient, the HE-stained slides originally used for diagnostic purposes were retrieved from the pathology archive and reviewed by the investigators, and those cases that were not classified by Lauren classification or were classified as mixed, were additionally reviewed and classified by a single expert gastrointestinal pathologist (T.J.K). Cancers of intestinal histological type were also graded for differentiation grade according to the WHO TNM classification, seventh edition^10^. The immutable national personal numbers assigned to each resident in the country were used to combine data from the patient records and the 100% complete follow-up data from the Causes of Death Registry at the Statistics Finland. Follow-up data were available until the end of 2016.

### Exposure (TSR)

The original, prospectively collected HE-stained diagnostic samples used for clinical decision-making were used for the present study. Multiple HE-stained sections from each patient were viewed with a light microscope, and a representative section with deepest invasion was used for further analysis. Sections were scanned and digitised using Aperio AT2 (Leica Biosystems, Wetzlar, Germany), and TSR was analysed from scanned slides using an Aperio ImageScope by two independent researchers (N.K. and M.E.) blinded to the clinical and outcome data.

TSR was analysed from invasive parts of tumour where most stroma was present, as areas with a high amount of stroma were considered to be decisive for prognosis based on previous results^6,7^. The part of tumour with most stroma was identified with a low magnification (×10–×50 total magnification). Single vision field at ×100 total magnification was used for analysis, and it was ensured that there were tumour cells present at all four corners of the vision field. Smooth muscle, mucin and necrosis were excluded from analysis, and the remaining area, consisting of tumour cells and stroma, was used. The area of stroma compared to the whole area under analysis was estimated and scored by 10% intervals. For example, a score of 20% meant that 20% of the analysed area consisted of stroma and 80% of the area consisted of tumour cells. As in the previously published literature, 50% ratio was defined as cut of point, and patients were divided into stroma-poor (proportion of stroma ≤50%) and stroma-rich (proportion of stroma >50%) groups^6,7^. When estimates differed by over 20% or were on the different sides of the 50% cut-off point, the slide was re-assessed with a third investigator (T.J.K.), an expert gastrointestinal pathologist, and consensus was reached.

### Outcomes

Primary outcome of the study was 5-year all-cause mortality, and the secondary outcome was overall all-cause mortality.

### Statistical analysis

Cohen’s kappa was calculated to analyse interobserver agreement. *χ*^2^-test was used to obtain *p*-values when comparing categorical variables. Continuous variables were compared, and *p*-values were obtained by *T*-test. The Kaplan–Meier method was used to compare mortality between groups, and the log-rank test was used to determine statistical significance of differences between groups. The Cox regression model was used to perform multivariable analysis, providing hazard ratios (HR) with 95% confidence intervals (CI). Cox regression was adjusted for confounding variables: (1) year of surgery (<2000 or ≥2000), (2) age at diagnosis (continuous variable), (3) sex (male or female), (4) administration of preoperative chemotherapy (yes or no), (5) tumour stage (stage I–II or stage III–IV), (6) Lauren classification (intestinal, diffuse, or mixed) and (7) radical resection (R_0_ or R_1/2_). Subgroup analyses were performed in Laurén intestinal, and diffuse-type gastric adenocarcinomas separately, adjusted for other confounders listed above, and also for histological grade (I–II or III) in the intestinal type subgroup. Sensitivity analysis was performed excluding patients with non-radical (R_1/2_) resection or distant metastases. The point estimates in the sensitivity analysis did not differ from the main analysis and therefore only the results of the main analysis are presented. IBM SPSS Statistics 24.0 (IBM Corp., Armonk, NY) was used for all statistical analyses.

## Results

### Patients

A total of 583 patients diagnosed with gastric carcinoma and undergoing surgical resection were included in the study. Median age of the patients was 69 years (range 27–90 years, interquartile range 15.4), with 352 (60.4%) of patients being men and 231 (39.6%) women. Only 22 (3.8%) of patients underwent preoperative chemotherapy. Of 583 patients, 437 (75.0%) underwent microscopically confirmed R_0_ resection and 146 (25.0%) had R_1/2_ resection. The patients with R_1/2_ resection included patients with non-curative intent, as well as 34 (5.8%) patients who had distant metastases at the time of surgery. Median follow-up time was 26 months (range 0–396 months).

### Assessment of TSR

TSR was successfully analysed for each patient and the patients were divided into stroma-poor (proportion of stroma ≤50%) and stroma-rich (proportion of stroma >50%) groups (Supplementary Fig. 1). No differences in the quality of the original glass slides from 1980s and 2010s were observed during the analysis. Total of 72 slides (12.3% of cases) needed re-assessment to reach consensus because of over 20% difference between the assessors or assessments on different sides of the cut-off point. Two major reasons for re-assessment were difficulties to find a representative vision field of the stroma-rich component fulfilling the condition of tumour cells being present at all sides of the vision field and the strong inflammatory reaction and reactive stromal cells that made it difficult to define the exact borders of tumour cell clusters. Cohen’s kappa value calculated before the re-assessment was 0.842, indicating good interobserver agreement between the two investigators.

Stroma-poor tumours were found in 241 (41.3%) of the patients, while 342 (58.7%) had stroma-rich tumours. In the stroma-rich group, there were proportionally more patients with surgery after the year 2000, younger age at diagnosis, high TNM stage, diffuse histological type and unradical resection (Table 1). High proportion of stroma was associated with high TNM stage in intestinal and diffuse-type subgroups and with unradical resection in the diffuse-type subgroup (Supplementary Table 1).

**Table 1:** Associations between tumour–stroma ratio and clinicopathological variables in 583 surgically resected patients with gastric adenocarcinoma

	Low proportion of stroma (*n* = 241)	High proportion of stroma (*n* = 342)	*P*-value
Year of surgery			0.005
≥2000	88 (36.5%)	165 (48.2%)	
<2000	153 (63.5%)	177 (51.8%)	
Mean age at diagnosis	69.3	65.0	<0.001^a^
Sex			0.17
Man	154 (63.8%)	198 (57.9%)	
Woman	87 (36.1%)	144 (42.1%)	
Preoperative chemotherapy			0.080
Yes	5 (2.1%)	17 (5.0%)	
No	236 (97.9%)	325 (95.0%)	
Tumour stage			<0.001
I–II	190 (78.8%)	169 (49.4%)	
III–IV	51 (21.2%)	173 (50.6%)	
Lauren classification			<0.001
Intestinal	174 (72.2%)	119 (34.8%)	
Diffuse	62 (25.7%)	208 (60.8%)	
Mixed	5 (2.1%)	15 (4.4%)	
Radicality of resection			<0.001
R0	205 (85.1%)	232 (67.8%)	
R1 or R2	36 (14.9%)	110 (32.2%)	

^a^*T*-test

### Primary outcome: 5-year mortality

**Fig. 1:**
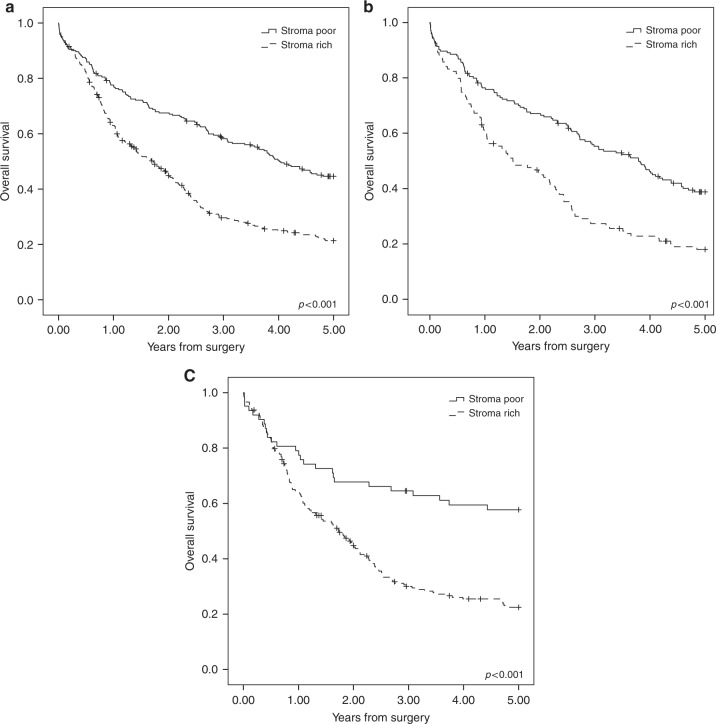
The Kaplan–Meier figures presenting 5-year survival stratified by the tumour–stroma ratio in gastric adenocarcinoma (**a**), 5-year survival stratified by the tumour–stroma ratio in the intestinal type gastric adenocarcinoma (**b**) and 5-year survival stratified by the tumour–stroma ratio in the diffuse-type gastric adenocarcinoma (**c**)

The 5-year prognosis was significantly better in the stroma-poor group (44.6%) compared to the stroma-rich group (21.3%, Fig. 1, *p* < 0.001, log-rank). In the univariable analysis, the stroma-rich group had significantly worse prognosis compared to the stroma-poor group (HR 1.94, 95% CI 1.57–2.40; Table 2). In multivariable analysis the stroma-rich group had significantly worse prognosis compared to the stroma-poor group (adjusted HR 1.80, 95% CI 1.41–2.28; Table 2).

**Table 2:** Univariable and multivariable analysis of the tumour–stroma ratio and prognosis in 583 patients with gastric adenocarcinoma

	Number of patients	Low proportion of stroma HR (95% CI)	High proportion of stroma HR (95% CI)
5-year mortality			
All patients (crude)	583	1.00 (Reference)	1.94 (1.57–2.40)
All patients (adjusted)	583	1.00 (Reference)	1.80 (1.41–2.28)^**a**^
*Subgroup analysis*			
Intestinal type (crude)	293	1.00 (Reference)	1.90 (1.43–2.51)
Intestinal type (adjusted)	293	1.00 (Reference)	1.68 (1.24–2.27)^b^
Diffuse type (crude)	270	1.00 (Reference)	2.48 (1.63–3.77)
Diffuse type (adjusted)	270	1.00 (Reference)	2.09 (1.35–3.23)^c^
Overall mortality			
All patients (crude)	583	1.00 (Reference)	1.75 (1.45–2.11)
All patients (adjusted)	583	1.00 (Reference)	1.71 (1.39–2.11)^a^
*Subgroup analysis*			
Intestinal type (crude)	293	1.00 (Reference)	1.75 (1.36–2.26)
Intestinal type (adjusted)	293	1.00 (Reference)	1,69 (1.29–2.21)^b^
Diffuse type (crude)	270	1.00 (Reference)	2.31 (1.62–3.28)
Diffuse type (adjusted)	270	1.00 (Reference)	2.01 (1.30–3.12)^c^

^a^Adjusted for year of diagnosis, age, sex, preoperative chemotherapy, tumour stage, Laurén classification and radical resection.

^b^Adjusted for year of diagnosis, age, sex, tumour stage, preoperative chemotherapy, radical resection and histological grade.

^c^Adjusted for year of diagnosis, age, sex, tumour stage, preoperative chemotherapy and radical resection

In the subgroup analysis, in patients with intestinal type histology, the 5-year prognosis was 38.8% in the stroma-poor group and 18.0% in the stroma-rich group (*p* < 0.001, log-rank). Similarly, in the multivariable analysis, the stroma-rich group had significantly higher 5-year mortality compared to the stroma-poor group (adjusted HR 1.68, 95% CI 1.24–2.27; Table 2). In the diffuse-type histology subgroup 5-year prognosis was 57.7% in the stroma-poor group and 22.4% in the stroma-rich group (*p* < 0.001, log-rank test). In multivariable analysis of the patients with diffuse-type histology, the stroma-rich group had significantly worse prognosis compared to the patients with stroma-poor tumours (adjusted HR 2.09, 95% CI 1.35–3.23; Table 2).

### Secondary outcome: overall mortality

In univariable analysis, patients with stroma-rich tumours had significantly higher mortality (HR 1.75, 95% CI 1.45–2.11) compared to the stroma-poor group (Table 2). In multivariable analysis, patients with stroma-rich tumours had significantly worse prognosis (HR 1.71, 95% CI 1.39–2.11) compared to the stroma-poor group (Table 2).

In the subgroup analysis of intestinal type tumours, the patients with stroma-rich tumours had significantly higher overall mortality compared to patients with stroma-poor tumours (adjusted HR 1.69, 95% CI 1.29–2.21; Table 2). In the diffuse-type histology subgroup, the patients with stroma-rich tumours had significantly worse prognosis compared to the patients with stroma-poor tumours (adjusted HR 2.01, 95% CI 1.30–3.12; Table 2).

## Discussion

The results of this study suggest that high proportion of stroma associates to poor prognosis in gastric adenocarcinoma, and similarly in both intestinal and diffuse histological subtypes of gastric adenocarcinoma.

There are some strengths and limitations that should be considered before interpreting the results. The present study is a retrospective single-institution study, which might limit its applicability for larger populations. However, the present study is the largest study on the association between TSR and mortality in gastric cancer and it is the only large study conducted in the Western countries. Another potential limitation of the study is the long study period from 1983 to 2016 during which the treatment of gastric adenocarcinoma patients has undergone major changes. However, the year of surgery was adjusted for in the multivariable analysis among other potential confounding variables, and the study period allowed long follow-up. The original diagnostic histological slides were used for evaluation and no differences in the quality between the old and new glass slides were observed. The low number of stroma-poor diffuse-type adenocarcinoma specimens limited the statistical power in the analyses of this subgroup.

In the present study, high proportion of stroma associated with the high TNM stage. Previous studies in gastric cancer have reported associations with high proportion of stroma and poor histological grade of differentiation^6,8,9^. As the non-radical resection was assumed to be associated with both TSR and mortality, patients with non-radical resection were included in the study to reduce selection bias and adjusted for in the analyses instead. The observed association between non-radical resection and high proportion of stroma could be explained with higher TNM stage of stroma-rich tumours, which can make radical resection more difficult or unachievable. High proportion of stroma was also associated with diffuse histological type, which might speculatively be related to the uncohesive and infiltrative growth pattern of diffuse gastric adenocarcinoma.

High proportion of stroma was associated with high 5-year mortality and high overall mortality, which is in line with the four previous studies on TSR and gastric cancer^6–9^. The previously largest cohort study on the topic (*n* = 494, from China) reported an HR of 1.91, with 95% CI 1.43–2.56, for the risk of death in the stroma-rich group of, but the study included only patients with complete histopathological and survival data available, making the selection bias apparent^9^. Another Chinese study (*n* = 225) suggested an HR of 2.75, with 95% CI of 1.86–4.07, for the 5-year mortality in the stroma-rich group in univariable analysis, but no adjusted analysis was presented for TSR, and the follow-up was based on telephone, mail and outpatient service^7^. A Korean study with 175 gastric signet-ring cell carcinomas showed an HR of 2.50 with 95% CI of 1.48–4.25 for 5-year survival in the stroma-rich group^6^. The only previous European study suggested an adjusted hazard ratio of 5.50, with 95% CI 2.34–12.92, but the study was conducted in a small (*n* = 106) and selected patient population, and the analysis was not adjusted, for example, for age at diagnosis, sex or histological subtype^8^. Compared to the previous studies, smaller hazard ratios for mortality comparing stroma-rich and stroma-poor groups were found in the multivariable analysis in the present study. These differences may be explained by number of factors. Three of the previous studies are from Asia^6,7,9^, where the aetiology of gastric cancer is different compared to the Western countries, with for example higher prevalence of *Helicobacter pylori* in the population^11^. In addition to differences in selection bias and demographics, the large proportion of diffuse-type tumours, and the inclusion of patients with R1–2 resection and distant metastases in the present study could also explain some of the differences in the estimates compared to the previous studies. However, the effect of R1–2 resection and metastasis on the results is not supported by the fact that results of the sensitivity analysis did not differ from the main analysis, and that the inclusion of patients with R1–2 resection and metastases should rather inflate the associations to mortality, not mitigate them. The subgroup analysis suggested that TSR is an independent prognostic factor for 5-year and overall mortality in both intestinal and diffuse types of gastric adenocarcinoma.

Currently it is not known why patients with higher proportion of stroma experience worse outcomes than those with low proportion of stroma. Probable mechanisms are based on interactions with tumour and stromal cells that have been subject to intense research in recent years^3,4,12^. CAFs have been found to mediate drug resistance of cancer^13^, promote angiogenesis^14^ and induce tumour cells to undergo epithelial–mesenchymal transition, which enhances invasive and metastatic capabilities of tumour^4^. They also contribute to immune evasion by tumour cells at least in some cancer types^15,16^. Accordingly, as stroma-rich tumours have more CAFs, they could reasonably benefit more of the growth supporting microenvironment CAFs create.

The results of the present study have clinical and research related implications. Our study provides further evidence on the prognostic value of TSR for gastric adenocarcinoma, also in the different histological subtypes of this malignancy. The analysis of TSR can be easily replicated and routinely analysed from HE-stained slides without additional immunohistochemistry or costs, making it easily applicable to clinical decision-making. Computer-assisted analysis for analysing the amount of stroma can be used, for example, in breast cancer^17^. Human assessment showed high interobserver agreement in the present study, but it would be useful to examine and validate computer-based assessment methods for gastric cancer in the future studies. Patients with low TSR might benefit from intensification of postoperative treatment, for example, chemoradiotherapy. Further typing of desmoplastic stroma on HE-stained classes has also been shown to distinguish high-risk patients in colorectal cancer^18,19^. It would be interesting to study its applicability in gastric adenocarcinoma, and whether taking different degrees of stromal maturation into account could increase the prognostic power of TSR. Prospective studies are needed to get more precise estimates of the prognostic value of TSR in gastric adenocarcinoma and its histological subgroups.

In conclusion, the results of this large study suggest that high proportion of stroma (low TSR) associates to poor prognosis in gastric adenocarcinoma, but its prognostic value might be lower than previously reported. High proportion of stroma is an independent prognostic factor in both intestinal and diffuse histological subtypes of gastric adenocarcinoma.

## Electronic supplementary material

Supplementary table 1

Supplementary figure 1

Supplementary figure legend

## Availability of data and material

We are willing to share anonymized data after the completion of the data collection upon request. Sharing the data will require ethical approval by the relevant committee, as well as approvals from the governmental agencies and local entities maintaining the relevant data.
