# Supplementary material for: Tumour-stroma ratio and prognosis in gastric adenocarcinoma
Source: Br J Cancer. 2018 Jul 30;119(4):435–9. doi: 10.1038/s41416-018-0202-y (PMC6133938; doi:10.1038/s41416-018-0202-y)
Supplement: Supplementary file 1 — Supplementary table 1 [file 41416_2018_202_MOESM1_ESM.docx]

**Supplementary Table 1.** Assocations between Tumour-stroma ratio and clinicopathological variables in intestinal and diffuse type histological subgroups of gastric adenocarcinoma.

|  | **Intestinal type** | | | **Diffuse type** | | |
| --- | --- | --- | --- | --- | --- | --- |
|  | **Low proportion of stroma**  **n = 174** | **High proportion of stroma**  **n = 119** | **P-value** | **Low proportion of stroma**  **n = 62** | **High proportion of stroma**  **n = 208** | **P-value** |
| **Year of surgery** |  |  | 0,54 |  |  | **0,020** |
| ≥2000 | 66 (37.9%) | 50 (42.0%) |  | 21 (33.9%) | 106 (51.0%) |  |
| <2000 | 108 (62.1%) | 69 (58.0%) |  | 41 (66.1%) | 102 (49.0%) |  |
| **Mean age at diagnosis** | 71.6 | 70.2 | 0.40^a^ | 63.6 | 62.0 | 0.64^a^ |
| **Sex** |  |  | 0.61 |  |  | 0.19 |
| Man | 114 (65.5%) | 82 (68.9%) |  | 38 (61.3%) | 107 (51.4%) |  |
| Woman | 60 (34.5%) | 37 (31.1%) |  | 24 (38.7%) | 101 (48.6%) |  |
| **Preoperative chemotherapy** |  |  | 0.28 |  |  | 0.53 |
| Yes | 3 (1.7%) | 5 (4.2%) |  | 2 (3.2%) | 12 (5.8%) |  |
| No | 171 (98.3%) | 114 (95.8%) |  | 60 (96.8%) | 196 (94.2%) |  |
| **Tumour stage** |  |  | **<0.001** |  |  | **<0.001** |
| I-II | 138 (79.3%) | 63 (52.9%) |  | 47 (75.8%) | 100 (48.1%) |  |
| III-IV | 36 (20.7%) | 56 (47.1%) |  | 15 (24.2%) | 108 (51.9%) |  |
| **Radicality of resection** |  |  | 0.063 |  |  | **0.002** |
| R0 | 149 (85.6%) | 91 (76.5%) |  | 52 (83.9%) | 130 (62.5%) |  |
| R1 or R2 | 25 (14.2%) | 28 (23.5%) |  | 10 (16.1%) | 78 (37.5%) |  |
| **Histological grade in intestinal type** |  |  | 0.15 |  |  | N/A |
| I | 33 (19.0%) | 14 (11.8%) |  | -- | -- |  |
| II | 82 (47.1%) | 54 (45.4%) |  | -- | -- |  |
| III | 59 (33.9%) | 51 (42.9%) |  | -- | -- |  |

^a^T-test
