# Supplementary figures and images for: Tumour-stroma ratio and prognosis in gastric adenocarcinoma
Source: Br J Cancer. 2018 Jul 30;119(4):435–9. doi: 10.1038/s41416-018-0202-y (PMC6133938; doi:10.1038/s41416-018-0202-y)

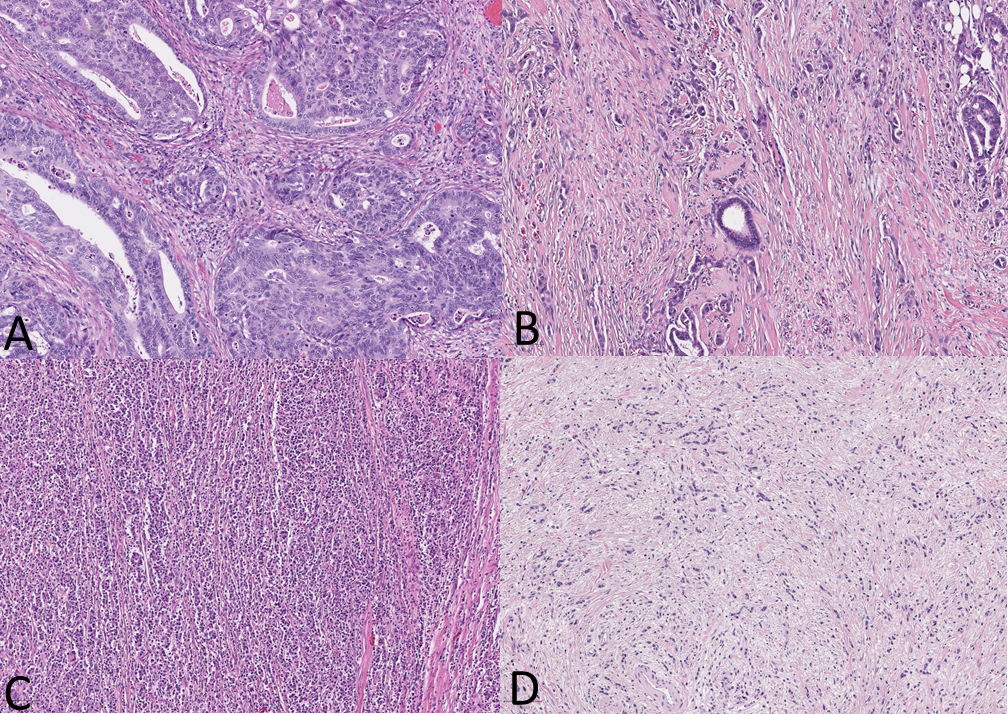

Supplement: Supplementary file 2 — Supplementary figure 1 [file 41416_2018_202_MOESM2_ESM.tif]
