# Supplementary material for: Tumour-stroma ratio and prognosis in gastric adenocarcinoma
Source: Br J Cancer. 2018 Jul 30;119(4):435–9. doi: 10.1038/s41416-018-0202-y (PMC6133938; doi:10.1038/s41416-018-0202-y)
Supplement: Supplementary file 3 — Supplementary figure legend [file 41416_2018_202_MOESM3_ESM.docx]

**Supplementary figure legends**

**Supplementary figure 1.** The photomicrographs show examples of low proportion of stroma in intestinal type cancer (A), high proportion of stroma in intestinal type cancer (B), low proportion of stroma in diffuse type cancer (C) and high proportion of stroma in diffuse type cancer (D), with 100x total magnification.
